# Honey Bee Colonies (*Apis mellifera* L.) Perform Orientation Defensiveness That Varies among Bred Lines

**DOI:** 10.3390/insects14060546

**Published:** 2023-06-12

**Authors:** Peter Njukang Akongte, Bo-Sun Park, Dong-Won Kim, Yong-Soo Choi

**Affiliations:** 1 Department of Agricultural Biology, National Institute of Agricultural Science, Wanju 55365, Republic of Korea; akongtepeter@korea.kr (P.N.A.); crambinae@korea.kr (B.-S.P.); dongwonkim@korea.kr (D.-W.K.); 2 Institute of Agricultural Research for Development (IRAD), Yaounde 2123, Cameroon

**Keywords:** honey bee bred lines, *Apis mellifera* L., controlled breeding, defensive behavior, chemical pheromone

## Abstract

**Simple Summary:**

Honey bee aggression constitutes an important defensive mechanism for honeybee colonies and humans. However, due to the complexity associated with this behavior, beekeepers are still faced with the challenges of selecting defensive and less-defensive bred lines. This could be achieved through selective breeding in closed mating stations. We endeavored to evaluate defensiveness and orientation in five bred lines of honeybee colonies using a combination of chemical (alarm pheromone and isopentyl acetate mixed with paraffin oil) and physical (dark leather suede, colony marbling, and suede jiggling) stimuli to identify defensive and less-defensive bred lines. We found that chemical assays associated with physical stimuli recruited bees to the suedes, but the time of recruitment was faster when stimulated by alarm pheromone compared to isopentyl acetate mixed with paraffin oil. Defensiveness varied among bred lines, and orientation was highest in defensive bred lines compared to less-defensive bred lines. Beekeepers are therefore advised to evaluate orientation defensiveness at the colony level and among bred lines when selecting breeding colonies for defensive purposes.

**Abstract:**

Honey bees (*Apis mellifera* L.) express complex behavioral patterns (aggressiveness) in defensive mechanisms for their survival. Their phenotypic expression of defensive behavior is influenced by internal and external stimuli. Knowledge of this behavior has recently become increasingly important, though beekeepers are still faced with the challenges of selecting defensive and less-defensive bred lines. Field evaluation of defensive behavior among bred lines of honey bees is required to overcome the challenges. Chemical cues (alarm pheromone and isopentyl acetate mixed with paraffin oil) and physical and visual stimuli (dark leather suede, colony marbling, and suede jiggling) were used to evaluate defensiveness and orientation among five bred lines of honeybee colonies. Our results showed that both chemical assays recruited bees, but the time of recruitment was significantly faster for alarm pheromone. Honeybees’ response to both assays culminated in stings that differed among bred lines for alarm pheromone and paraffin when colonies were marbled. Honeybee orientation defensiveness varied among bred lines and was higher in more defensive bred lines compared to less-defensive bred lines. Our findings suggest that it is crucial to repeatedly evaluate orientation defensiveness at the colony level and among bred lines when selecting breeding colonies.

## 1. Introduction

Among social organisms, honey bees exhibit defensive mechanisms as a fundamental tool for their survival [1]. This complex behavioral pattern (defensive behavior) is expressed in *Apis mellifera* L. and influenced by external and internal stimuli [2,3], which may vary in similar colonies in an apiary [4,5,6]. The phenotypic expression of behavioral patterns in honeybee colonies could depend on both individual and colony characterization [7,8]. The survival and production of hive products in honeybee colonies depend on their behavioral output, which is a key factor in animal ecology. For instance, the removal of diseased brood in honeybee colonies is attributed to hygienic behavior [9,10]; good communication network, pollen, and honey storage are eased by wax and comb-building ability [11]; and protection of colony resources is aided by aggressive behavior [1]. 

Defensiveness in honey bees is not only beneficial to honeybee colonies but to humans as well. Although aggressive bees have been known to be a potential source of death to animals and humans [3], keeping significantly defensive honeybee colonies has become a conservation-positive activity [12]. Knowledge of honeybee defensiveness has recently become very important regardless of the public hazards associated with their painful stings. Honey bee stings are well known to deter a wide range of intruders (predators) around honeybee colonies. Using *Apis mellifera* in beehive fences [13,14,15,16] is even known to be a successful means of managing crop raiding by elephants [13,17].

Aggression in honeybee colonies is affected by many factors. However, the release of stings spreads pheromones that mitigate their defensive behavior to culminate in a massive attack [18]. In another study, aggression is performed by worker bees defending their hives against intruders [3,19] and is facilitated by a massive response coordinated by many worker bees [3]. Additionally, social information due to environmental changes could impact adult bee responsiveness to aggressive cues [20], which further regulate individual phenotypes displayed by nest mates [21,22,23]. 

Defensiveness in honey bees has been reported as a complicated behavior with well-defined components (environmental, maturity, and inherited) [3,24]. It is thought to be fully developed in bees older than 7 days [25], and its phenotypic expression varies among species. For instance, Africanized bees were reported to be more defensive than European bees [26,27,28]. Of the 31 known subspecies, the subspecies Apis mellifera mellifera, naturally distributed in Western and Northern Europe, is characterized by the most pronounced defensive behavior, which developed in the process of coevolution with bears and positively correlates with the honey productivity of colonies [29]. This trait was also found to be heritable [30] and genetically dominant [31,32]. In the past decades, field investigations have been carried out to evaluate the defensive behavior in *Apis mellifera*; meanwhile, it is necessary to have a better understanding of the phenotypic variability in defensiveness among bred lines of honeybee colonies. Important drivers of defensiveness(pursuit and stinging) that are highly linked to orientation flight are considered to determine this aspect. 

The demand for defensive bees by humans has called on the attention of honeybee breeders to select and breed defensive bees for a purpose. However, defensive bees can pursue and sting any moving object upon disturbance [33] without targeting. The use of chemical pheromones [34] and small moving objects [22,34] are field assays adopted to evaluate defensiveness in honey bees. However, knowledge of honeybee orientation defensiveness towards small moving targeted objects, when disturbed, is scant. Thus, bees that are capable of orientating their aggressiveness towards small, targeted objects when disturbed are of great interest. To achieve this, field evaluation of defensiveness among bred lines of honey bees is required to overcome the challenges by practicing controlled breeding to maintain desirable traits [35]. We bred *Apis mellifera* L. in isolated mating stations and used field assays to simultaneously evaluate their defensive responses. We repeatedly examined whether defensiveness is oriented and differs among bred lines as well as the minimum time within which honeybees’ response to this behavior is clearly visible. This study provides evidence that defensiveness and orientation vary among bred lines of honeybee colonies and that defensive bees oriented their flight more than less-defensive bees. 

## 2. Materials and Methods

### 2.1. Breeding and Placement of Honey Bee Colonies at the Experimental Apiary

Honeybee colonies of *Apis mellifera* L. were selected in early spring (March) 2022 from the experimental apiary (35.591° N, 126.278° E) of the honeybee breeding laboratory of the National Institute of Agricultural Sciences, Rural Development Administration, Wanju, Republic of Korea. The colonies were made up of five lineages (bred lines) of *Apis mellifera* L. (lines A, B, C, D, and E). Some characteristics of these bred lines are presented in Table S1. In spring (April to May 2022), a single strong queenright colony was selected from each lineage for drone and queen rearing. One empty built comb and one empty drone comb were marked and provided to each colony for the queen to deposit fertilized and unfertilized eggs, respectively. These combs were inserted in-between broods, and the presence of eggs was checked every 24 h. Within a period of 3 days, when eggs are expected to hatch into larvae, the queen cell combs were removed to transfer the first-instar (≤20 hold) larvae into artificial queen cell cups for queen rearing [36]. Each queen cell cup attached to rearing frames contained one drop (5 mL syringe) of diluted royal jelly in water at a ratio of 1:1 (*v*/*v*). The queen bee was excluded with a queen excluder by making a second floor (supper) for rearing the larvae. Each colony was fed sugar syrup and pollen cake as food supplements for drone and queen rearing. Ten mating nuclei (mating hives) were prepared for each lineage. Each mating hive contained one brood comb with worker bees, one food comb (honeycomb), a feeder, and 2–3 capped queen cells. Regarding the life cycle of queen (16 days) and drone (25 days) bees, drones were reared and taken to mating stations before mating hives. Three days prior to queen emergence, 2–3 capped queen cells were inserted into each mating hive and placed at mating stations. The presence of emerged queens was examined three days later. After monitoring mating hives at mating stations for fifteen days, we recorded the presence of eggs and larvae. Successfully mated queens were moved with nuclei hives to the experimental apiary. Each lineage was bred separately from one another in a closed mating system. Although mating success was not 100%, mated colonies were placed at the experimental apiary according to lineage (bred lines). Colonies were fed sugar syrup and pollen cake for colony development, after which experimental colonies were selected per bred line. 

### 2.2. Chemical, Physical, and Visual Stimuli Application (Assays)

We applied a series of assays according to Collins et al. [37] to test the defensive behavior in honey bee colonies. Chemicals associated with the honey bee sting (alarm pheromones) were mixed and diluted in paraffin oil at a ratio of 1:9 (*v*/*v*) as described by Blum et al. [38] with the omission of n-decyl acetate (Table 1). To provide a visual stimulus, a square of dark suede leather (5 cm by 5 cm) made from animal skin (pig) (Figure S1) was attached to movable objects and jiggled at the colonies’ entrances. Honey bee colonies were struck with a standard marble stimulus from behind as a physical stimulus to elicit aggressiveness. A combination of these stimuli was provided to each colony to evaluate their defensive behavior.

### 2.3. Assessment of Defensiveness and Orientation in Honey Bee Colonies 

Fifteen queenright colonies from five bred lines (three colonies per bred line) placed at the experimental apiary were selected and used for this study. Sampling was carried out in autumn (September and October, late flowering season), during the day, and at an interval of two days. The experiment was conducted in two parts: evaluation of defensive behavior (experiment 1) and determination of orientation defensiveness (experiment 2).

Experiment 1: To evaluate the defensive behavior in the honeybee colonies, three treatments were employed to test defensiveness in honey bees: T1, alarm pheromone (Table 1); T2, chemical composition of isopentyl acetate mixed with paraffin oil at a ratio of 1:9 (*v*/*v*); and T3, no chemical application. Johnson et al. [39] reported that isopentyl acetate (IPA) is the principal component of the alarm pheromone. T1 and T2 were sprayed on square suedes, while T3 were empty suedes (suedes that did not have a chemical sprayed on them) (control). In this experiment, colonies were not disturbed (no marbling). Suedes were attached to movable objects and held 40 cm perpendicular to the hive entrance. Movable objects with suedes were jiggled vertically at 2 rounds per second(rps) to provide a visual cue to bees. Sampling was carried out once a day at 10 s, 30 s, 60 s, and 90 s using a stopwatch at an interval of two days and replicated four times. In this experiment, each bred line was replicated three times, making a total of 15 colonies for the 5 bred lines. Each colony per bred line was repeatedly tested four times at 10 s, 30 s, 60 s, and 90 s with T1, T2, and T3. Each bred line was tested 4 times per colony, per assay, and per duration (10 s, 30 s, 60 s, and 90 s). We recorded the time for the first attack on the suede, the culminating intensity, and the number of stings left on the suede. The degree of crowdedness of honey bees on the suede was evaluated and recorded as high (when suedes were fully covered with bees over the course of the test), average (when suedes were not fully covered with bees over the course of the test), and very low (when very few bees or no bees were found on the suede over the course of the test). These scores were recorded during each sampling, and the frequency of occurrence of a score was used as a positive grade to determine the intensity of recruitment per assay. The dark leather suede used in this study was obtained from Future Technic Co., Ltd. (Bangkok, Thailand) (www.sciencetool.co.kr (accessed on 1 June 2023)).

Experiment 2: To determine whether honey bee colonies perform orientation defensiveness, alarm pheromone (Table 1) and paraffin oil were sprayed on separate square leather suedes using a hand sprayer (Apollo Ind Co. Ltd., Gyeonggi-do, Republic of Korea). Both suedes were attached to movable objects and held 40cm perpendicular to the hive entrance and 30 cm apart. Movable objects with suedes were jiggled up and down simultaneously at 2 rps to provide a visual cue to bees. Paraffin was used as the control. The experiment was conducted in two rounds (when colonies were struck with a standard marble stimulus and when colonies were not struck with a standard marble stimulus). We anticipated that the simultaneous introduction of alarm pheromone and paraffin would be a positive test to determine orientation when colonies are disturbed (struck with a standard marble stimulus) and when normal (not disturbed or not struck with a standard marble stimulus or docile). Cues necessary for alerting, activating, recruiting, and attacking (chemical pheromone sprayed on dark leather suedes attached to movable objects and jiggled vertically at 40 cm perpendicular to hive entrance) were provided to provoke a response. Colonies were struck with a standard marble stimulus once during each sampling to allow colonies to gain stability before the next sampling. Sampling was carried out in 10 s, 30 s, 60 s, and 90 s durations, with each length of time being replicated four times. In this experiment, each bred line was replicated three times, making a total of 15 colonies for the 5 bred lines. Each colony per bred line was repeatedly tested four times at 10 s, 30 s, 60 s, and 90 s with alarm pheromone and paraffin. In each bred line, we tested 4 times per colony, per assay (alarm pheromone and paraffin), and per duration (10 s, 30 s, 60 s, and 90 s). This experiment was conducted in two phases (when colonies were struck with a standard marble stimulus and when colonies were not struck with a standard marble stimulus). The number of stings left on the suede was recorded in all instances. 

### 2.4. Data Analysis 

Data were described using Descriptive Statistics. One-way analysis of variance (ANOVA) was used to compare the means of more than two groups, followed by the Tukey (HSD)post hoc test for multiple pairwise comparisons of variance. Two-tailed Student’s *t*-test and the Mann–Whitney test were used to compare the means of two groups. Pearson’s correlation was used to evaluate the relationship between the mean number of stings for alarm pheromone and paraffin mixed with IPA over the experimental period. The non-parametric Kruskal–Wallis test, followed by multiple pairwise comparisons of variance using Dunn’s procedure, was used to compare the means of more than two groups that were not normally distributed. The XLSTAT statistical software version 2007.8.04 was used to conduct the analysis with levels of significance set at 5%.

## 3. Results

### 3.1. Experiment 1: Evaluating the Defensive Behavior of Honey Bees among Bred Lines 

#### 3.1.1. Effects of Assay (Alarm Pheromone, Isopentyl Acetate/Paraffin, and Empty Suedes) on Time and Intensity of Recruitment of Honey Bees on the Suede 

The recruitment time of honey bees towards an assay was used to describe the level at which respective assays could alert bees under the same visual cue and environmental conditions. The degree of the crowdedness of honey bees around assays showed the intensity of the alert. The intensity of recruitment varied from high (for alarm pheromone) to average (for isopentyl acetate/paraffin) to low (for the empty suede) (Table 2). Using the alarm pheromone test, the time of recruitment of honey bees was marginally significant among bred lines (F_4,235_ = 2.386, *p* = 0.052) (Table 2). Meanwhile, the time of recruitment varied significantly among bred lines when a mixture of paraffin and IPA was applied (F_4,235_ = 12.063, *p* < 0.0001) (Table 2). The time of recruitment for the control (empty suedes) did not differ significantly among bred lines (F_4,92_ = 0.105, *p* = 0.981) (Table 2). Within bred lines, the time of recruitment equally differed significantly between the three assays: line A (F_2,116_ = 79.457, *p* < 0.0001); line B (F_2,111_ = 86.603, *p* < 0.0001); Line C (F_2,116_ = 62.636, *p* < 0.0001); line D (F_2,105_ = 56.180, *p* < 0.0001); and line E (F_2,114_= 73.332, *p* < 0.0001). 

**Figure 1:**
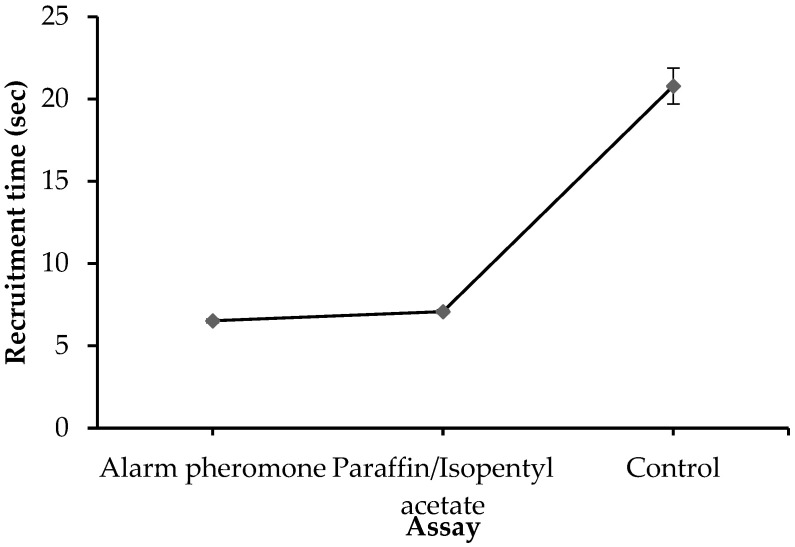
Effect of different assays on recruitment time for all bred lines. Error bars are mean ± SE.

Generally, the time of recruitment varied significantly among assays (F_2,56_ = 56.955, *p* < 0.0001). The mean time of recruitment for alarm pheromone, paraffin mixed with IPA, and the control was 6.52 s, 7.08 s, and 18.88 s, respectively (Figure 1). Similarly, the time of recruitment recorded for alarm pheromone and paraffin mixed with IPA differed significantly (t = 1.965, *p* = 0.0004). However, the time difference in recruitment between alarm pheromone and paraffin mixed with IPA was relatively small (0.56 s) compared to that of the control (12.36 s) (Figure S2). 

#### 3.1.2. Behavioral Responses of Honey Bee Colonies to Different Assays

**Figure 2:**
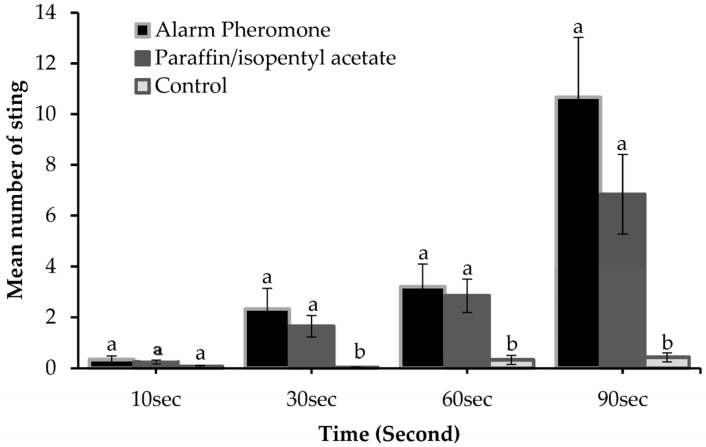
Honey bee response (number of stings) to assays at different time intervals for all bred lines. Error bars are mean ± SE. Means with different small letters at respective times indicate a significant difference between the assays; *p* < 0.05, one-way ANOVA, Tukey (HSD)post hoc test.

The behavioral response of honey bees, which culminated in a sting, varied according to assay and time of sampling (Figure 2). The results indicated that the mean number of stings for the three assays did not differ significantly at 10 s (F_2,42_ = 1.799, *p* = 0.178) but differed significantly at 30 s (F_2,42_ = 4.577, *p* = 0.016), 60 s (F_2,42_ = 5.082, *p* = 0.011), and 90 s (F_2,42_ = 6.983, *p* = 0.002) (Figure 2). Though no significant difference was found for the mean number of stings between alarm pheromone and paraffin mixed with IPA, it is evident that the mean number of stings was higher for alarm pheromone suedes compared to paraffin mixed with IPA suedes in all instances (Figure 2). This indicates that honey bees responded more to the alarm pheromone suedes than other suedes. The duration at which assays were exposed to honey bee colonies influenced their stinging response as the number of stings increased with the time of exposure (Figure 2).

**Figure 3:**
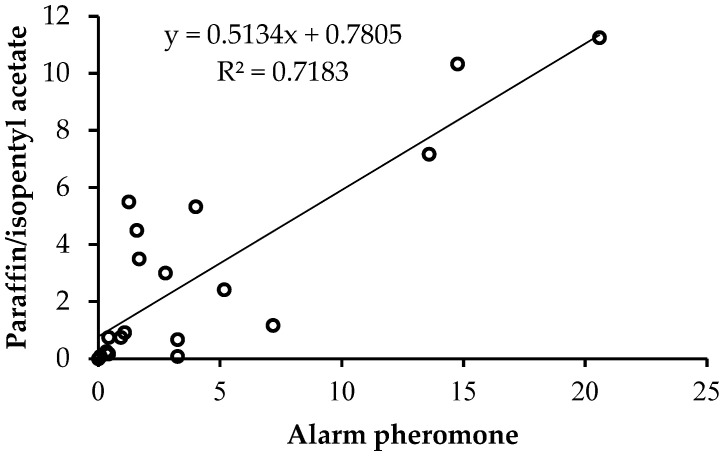
Pearson’s correlation between the mean number of stings for alarm pheromone and paraffin mixed with IPA for the five bred lines sampled from 10 to 90 s. The circle represent the correlation.

Honey bees responded positively to the two chemical assays, but the rate of response was higher in response to alarm pheromone than paraffin mixed with IPA at all intervals (Figure 2). The mean number of stings for both chemical assays increased with increasing time of exposure. However, the degree of increase was higher in response to alarm pheromone suedes compared to those of paraffin mixed with IPA (Figure 3). A significantly positive correlation was recorded between honey bee response (number of stings) to the two chemical assays at different times of sampling (r = 0.848, *p* < 0.0001) (Figure 3). 

#### 3.1.3. Variation in the Aggressive Behavior among Honey Bee Bred Lines 

The mean number of stings did not vary significantly in lines A, B, C, and D but varied significantly in line E for both the alarm pheromone and paraffin mixed with IPA (Table 3). In the control experiment, the mean number of stings per line did not differ significantly for all five bred lines (Table 3). We found a significant difference in the mean number of stings among bred lines for alarm pheromone and control but not for paraffin mixed with IPA (Table 3). Generally, honey bee defensiveness was significantly higher in lines E and C compared to other bred lines across all three treatments. However, no significant difference was observed per bred line between alarm pheromone and paraffin mixed with IPA. This indicates that honey bee aggression, which culminated in a sting, was similar to the alarm pheromone and paraffin mixed with IPA. Though in most bred lines (80%), the mean number of stings was higher in the alarm pheromone compared to paraffin mixed with IPA, paraffin mixed with IPA is a chemical cue necessary to elicit a defensive response in honey bees.

### 3.2. Experiment 2: Evaluating Orientation Defensiveness among Honey Bee Bred Lines

#### Orientation Aggressiveness among Bred Lines of Honey Bee Colonies

**Figure 4:**
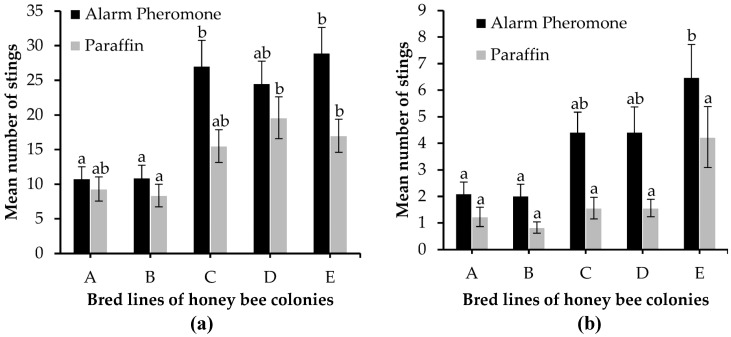
Orientationdefensivenessin honey bee bred lines in response to external stimuli. (**a**) When colonies were struck with a standard marble stimulus, and(**b**)when colonies were not struck with a standard marble stimulus. Error bars are mean ± SE. In (**a**) and (**b**), means with different small letters on the same treatment (alarm pheromone, paraffin) differ significantly among bred lines. *p* < 0.05, Kruskal–Wallis test using Dunn’s procedure.

The mean number of honey bee stings in response to either alarm pheromone or paraffin suedes was recorded when colonies were struck with a standard marble stimulus and when colonies were not struck with a standard marble stimulus (Figure 4). Colonies C, D, and E were found to be the most defensive and orientated their flight towards the alarm pheromone suedes (Figure 4). We found significant differences in the mean number of stings between alarm pheromone and paraffin when colonies were struck with a standard marble stimulus (U = 33,109, *p* = 0.004) and when colonies were not struck with a standard marble stimulus (U = 36,683, *p* < 0.0001). Generally, the mean number of stings for alarm pheromone was higher than that of paraffin when colonies were struck with a standard marble stimulus (20.44 ± 1.42 and 13.96 ± 1.05, respectively) and when colonies were not struck with a standard marble stimulus (3.89 ± 0.38 and 1.88 ± 0.28, respectively) (±SE). When colonies were struck with a standard marble stimulus, the mean number of stings differed significantly among bred lines for alarm pheromone (K = 25.185, df = 4, *p* < 0.0001) and paraffin (K = 14.551, df = 4, *p* = 0.006). This did not follow the same scenario when colonies were not struck with a standard marble stimulus because the mean number of stings varied significantly among bred lines for alarm pheromone (K = 16.178, df = 4, *p* = 0.003) but insignificant for paraffin (K = 9.211, df = 4, *p* = 0.056) (Table S2).

**Figure 5:**
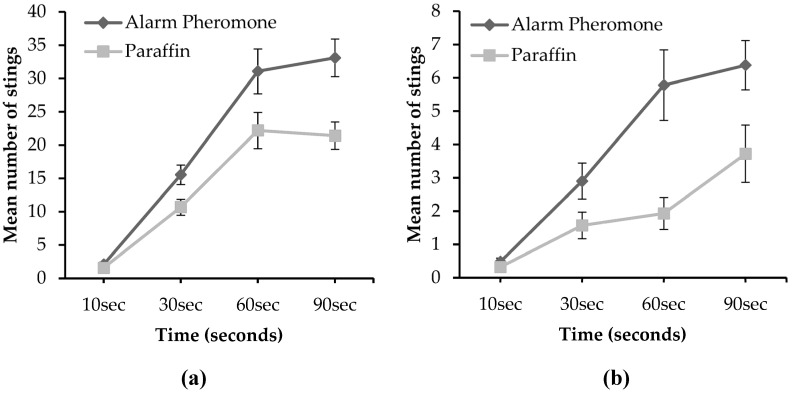
Honeybees’ orientation defensiveness in response to external stimuli with respect to time of exposure. When colonies were struck with a standard marble stimulus (**a**) and when colonies were not struck with a standard marble stimulus (**b**). Error bars are mean ± SE.

In line A, there was no significant difference between the mean number of stings for alarm pheromone and paraffin when colonies were struck with a standard marble stimulus (U = 1258, *p* = 0.438) and when colonies were not struck with a standard marble stimulus (U = 1387, *p* = 0.063). The mean number of stings did not differ significantly between alarm pheromone and paraffin when colonies were struck with a standard marble stimulus (U = 1275, *p* = 0.363) but differed significantly when colonies were not struck with a standard marble stimulus (U = 1456, *p* = 0.016) in line B. We recorded significant differences in the mean number of stings between alarm pheromone and paraffin when colonies were struck with a standard marble stimulus (U = 1467, *p* = 0.021) and when colonies were not struck with a standard marble stimulus (U = 1611.5, *p* = 0.0004) in line C. In line D, the mean number of stings did not differ significantly between alarm pheromone and paraffin when colonies were struck with a standard marble stimulus (U = 1280, *p* = 0.348) but differed significantly when colonies were not struck with a standard marble stimulus (U = 1443.5, *p* = 0.027). The mean number of stings between alarm pheromone and paraffin in line E differed significantly when colonies were struck with a standard marble stimulus (U = 1432, *p* = 0.04) and when not struck with a standard marble stimulus (U = 1454, *p* = 0.024). However, no significant differences were observed between the mean number of stings for alarm pheromone (when colonies were struck with a standard marble stimulus, U = 1658, *p* = 0.457, and when not struck with a standard marble stimulus, U = 1472.5, *p* = 0.084) and paraffin (when struck with a standard marble stimulus, U = 1727.5, *p* = 0.705,and when not struck with a standard marble stimulus, U = 1470, *p* = 0.071) at 60 s and 90 s (Figure 5).

## 4. Discussion

Honey bee workers express behavioral responses to defend their hives against intruders (defensive behavior) [3] and equally remove dead broods and parasites and limit the spread of some diseases in the colonies [40].

Though we found a significant difference in the time of recruitment between alarm pheromone and paraffin mixed with IPA, a mixture of paraffin and IPA recruited honey bees within the average possible time (Figure S3). Our results agree with those of Wager and Breed [41], who found that IPA led to a higher level of flight activity and recruited bees more than other components of the alarm pheromone. The present study supports the fact that IPA is a major component of the sting alarm pheromone [42,43]. However, different concentrations of IPA mixed with paraffin oil and their effect on the degree and intensity of recruitment of honey bees still need to be investigated because Lenskyet al. [44] reported that increasing levels of stinging responses of isolated bees correspond to the concentration of IPA used for stimulation. A more recent study has demonstrated that for lone bees, the efficacy of recruitment depends on the alarm pheromone level [45] though they cannot re-assess their decision after the threshold level of the pheromone.

Despite the combination of many chemicals to form the alarm pheromone, IPA showed the capacity to elicit defensive responses (alerting, recruiting, and attracting) in honey bees which also increased with time of exposure. Collins et al. [37] reported that IPA is probably a stimulus to elicit defensive behavior in honey bees by alerting, activating, and attracting the bees. In 1989, Collins et al. [43] found positive correlations between levels of IPA and defensive behavior in the field (number of stings) and in the laboratory. Collins and Kubasek [34] documented that the mean number of bees responding to the chemical pheromone outside the colony entrance increases with time. The marginally significant difference in the number of stings between alarm pheromone and paraffin mixed with IPA could be attributed to the multi-component of the alarm pheromone, which provided a complex signaling system to the bees. According to the results obtained by Petrov et al. [46], they hypothesized that aggressiveness in honey bees increases linearly with pheromone level.

In most bred lines (80%), the mean number of stings was higher in the alarm pheromone compared to paraffin mixed with IPA (paraffin mixed with IPA is a chemical cue necessary to elicit a defensive response in honey bees). According to a model of honey bee defensive behavior [37], IPA is probably a stimulus for alerting, activating, and attracting but not for culminating. In this study, it is evident that honey bee response to paraffin mixed with IPA culminated in a sting. In all apiaries and in the wild, honey bee colonies respond aggressively when disturbed or towards a stimulus, but the intensity of the response may differ from colony to colony [21]. Defensiveness in honey bees is a social behavior well known to be influenced by environmental, maturational, and hereditary factors [3,24]. In another study carried out by Bianchi et al. [47], the defensive behavior of honey bees (fly and sting) showed significant differences between apiaries from different locations. The variation in our study (among bred lines) could be linked to the characteristics of the bred lines because environmental and maturational factors were kept constant. We anticipated that the duration of exposure of honey bees to paraffin mixed with IPA could orientate their defensiveness and, consequently, the number of stings. Harrison et al. [48] reported that long-term modulation of social cues induced changes in the phenotypic expression of aggression.

Defensive bred lines showed a high potential of performing orientation flight which culminated in a sting when colonies were disturbed than less-defensive bred lines. The simultaneous introduction of alarm pheromone and paraffin on suedes at the hive entrance induced a cue that permitted bees to orientate their flight. Other methods, such as video recording, are also used to track flight paths at the hive entrance [49]. Scheineret al. [50] used behavioral assays to induce a response by disturbing the hive. It is still uncertain whether honey bees can orientate their flight by distinguishing two nearby cues when disturbed. Collins and Kubasek [34] reported that 90% of honey bees responded to the alarm pheromone within an average time of 13.6 ± 8.95 s. Bochet al. [51] found that the capacity of the alarm pheromone to alert and attract bees at the hive entrance was 1.32 times greater than that of identical concentrations of IPA though both assays were not simultaneously introduced. This study indicates that honey bees orientated their defensive behavior towards the alarm pheromone more than the control (paraffin) in both cases (when colonies were struck with a standard marble stimulus and when colonies were not struck with a standard marble stimulus). However, the degree of orientation varies with bred lines and defensiveness, where very defensive bred lines performed orientation more than less-defensive lines. For instance, bred lines C, D, and E expressed a high level of defense and orientated their defensiveness toward a particular stimulus (alarm pheromone) compared to bred lines A and B with a low level of defense (Table S2). Again, we recorded insignificant disparity for orientation defensiveness within individual bred lines of honeybee colonies. Hunt et al. [24] reported that defensiveness in honey bees is influenced by hereditary factors. It is necessary to understand the extent to which the phenotypic expression of the hereditary factors varies among different bred lines of the same species. In another study conducted by Harrison et al. [48], the phenotypic expression of defensiveness in honey bees can be modulated due to differentiation in social cues by certain genes over the course of time. In our study, the phenotypic variation in the defensive behavior among bred lines of honey bee colonies could be attributed to the characteristics of the different bred lines.

## 5. Conclusions

Honey bee defensiveness is a social behavior that can be influenced by internal and external cues. In this study, the results demonstrated that both alarm pheromone and paraffin mixed with IPA recruited bees to suedes, but the time of recruitment was faster when the alarm pheromone was used compared to paraffin mixed with IPA. Honey bee response to both pheromones culminated in a sting, though it was greater when the alarm pheromone was used. The phenotypic expression of defensiveness varied among bred lines of honey bee colonies. Honey bees performed orientation defensiveness which varied among bred lines and was higher in more defensive bred lines compared to less-defensive bred lines. Therefore, it is crucial to repeatedly evaluate defensiveness and orientation at the colony level and among bred lines when selecting defensive and less-defensive honey bee colonies for breeding. Further studies should be conducted to evaluate the different concentrations of IPA mixed in paraffin necessary to elicit defensiveness and orientation in honey bee colonies within the shortest possible time. The use of a single chemical to determine defensive bees is less costly compared to the complex alarm pheromone if similar results could be obtained.

## Figures and Tables

**Table 1:** Chemical composition of the alarm pheromone.

Chemical Type	Volume (mL)	Volume (µL)	Percentage Composition (%)
n-Butyl acetate	0.15	150	1.5
Isopentyl acetate	3.2	3200	32
Isopentyl alcohol	1.5	1500	15
n-Hexyl acetate	0.4	400	4
n-Octyl acetate	1.7	1700	17
2-Nonanol	1.0	1000	10
Benzyl acetate	1.6	1600	16
Benzyl alcohol	0.45	450	4.5
Paraffin oil	90	90,000	900
	Total = 100 mL	100,000 (µL)	1000

**Table 2:** Variation in recruitment time and intensity of recruitment among honey bee bred lines.

Bred Lines	Chemical Assay		Control
	Alarm Pheromone (Time/s) (Mean ± SE)	Paraffin/Isopentyl Acetate (Time/s) (Mean ± SE)	Empty Suedes (Time/s) (Mean ± SE)
A	6.48 ± 0.23 Aa	7.79 ± 0.21 Aa	21.43 ± 2.26 Ab
B	6.77 ± 0.26 Aa	7.87 ± 0.26 Aa	21.06 ± 2.2 4Ab
C	6.1 ± 0.24 Aa	7.23 ± 0.24 ABa	19.56 2.28 Ab
D	7.02 ± 0.27 Aa	6.58 ± 0.22 BCa	21.5 ± 3.78 Ab
E	6.21 ± 0.24 Aa	5.94 ± 0.25 Ca	20.81 ± 2.5 Ab
Recruitment intensity	High	Average	Very low

Means within a column having the same capital letter shows no significant difference among bred lines and means within rows followed by different small letters are significantly different between assays for each bred line at *p* < 0.05, one-way ANOVA, Tukey (HSD) post hoc test.

**Table 3:** Mean number of stings among bred lines of honey bee colonies.

Bred Lines	Alarm Pheromone			Paraffin/Isopentyl Acetate			Control (No Chemical)		
	No. of Stings (Mean ± SE)	F-Value	*p*-Value	No. of Stings (Mean ± SE)	F-Value	*p*-Value	No. of Stings (Mean ± SE)	F-Value	*p*-Value
A	2.21 ± 0.77 ab	0.462	0.633	2.62 ± 0.84 a	0.136	0.873	0.12 ± 0.07 abc	1.898	0.162
B	1.08± 0.56 b	0.586	0.561	1.04 ± 0.37 a	1.23	0.302	0.02 ± 0.02 c	1	0.376
C	5.69± 1.75 ab	1.152	0.325	3 ± 1.14 a	0.86	0.43	0.46 ± 0.25 a	0.451	0.64
D	4.69± 1.91 ab	0.469	0.629	2.85 ± 1.43 a	0.317	0.73	0.04 ± 0.03 bc	0.5	0.61
E	6.98± 2.12 a	3.655	0.034	4.98 ± 1.07 a	3.368	0.043	0.4 ± 0.15 ab	0.07	0.932

In a column, means with different small letters differ significantly among bred lines; *p* < 0.05, one-way ANOVA, Tukey (HSD) post hoc test.

## Data Availability

The data presented in this study are within the manuscript and available on request from the corresponding author.

## Supplementary Materials

The following supporting information can be downloaded at: https://www.mdpi.com/article/10.3390/insects14060546/s1. Table S1: Characteristics of bred lines used in this study; Table S2: Variation in the mean number of stings among bred lines when colonies were struck with a standard marble stimulus and when colonies were not struck with a standard marble stimulus; Figure S1: Dark leather suedes used for evaluating defensiveness in honey bees; Figure S2: Box plot for the time of recruitment of honey bee per assay; Figure S3: Variation in the recruitment time among bred lines of honey bee colonies.
