# Supplementary material for: Honey Bee Colonies (Apis mellifera L.) Perform Orientation Defensiveness That Varies among Bred Lines
Source: Insects. 2023 Jun 12;14(6):546. doi: 10.3390/insects14060546 (PMC10298944; doi:10.3390/insects14060546)
Supplement: Supplementary file 1 [file insects-14-00546-s001.zip › insects-2414270-supplementary.pdf]

Supplementary materials

Table S1. Characteristic of bred lines used in this study.

Honey bee bred lines	Characteristics of individual bred lines
A	-Gentle
B	-High honey production<br>-Hygienic<br>-Good comb building and high propolis production
C	-Rapid colony development<br>-High fertility
D	-High royal jelly production<br>-Overwintering activity
E	-High pollen collection<br>-High royal jelly production<br>-Hygienic

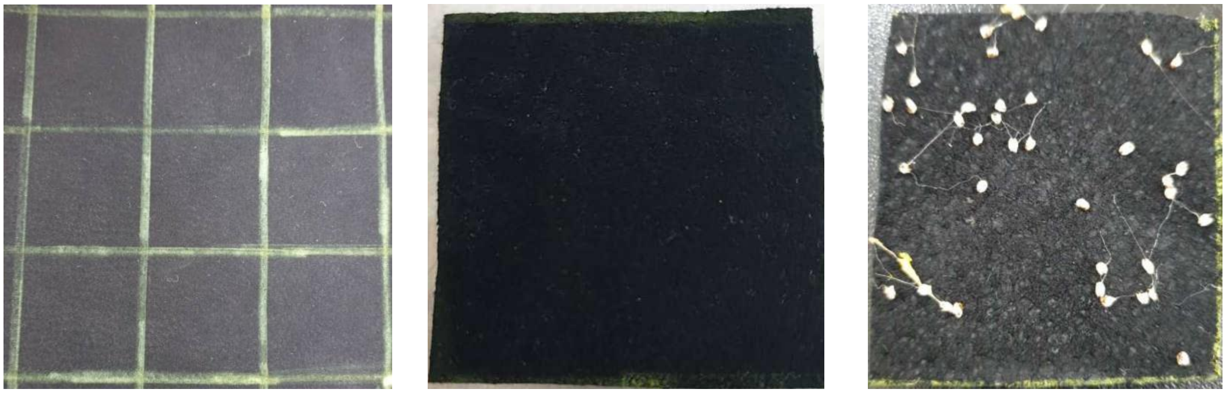

(a) (b) (c)

Figure S1: Dark leather suedes used for evaluating defensiveness in honey bees. Marked leather suede in squares of 5 by 5 cm (a), square leather suede treated with chemical assays (b), square leather suede containing honey bee stings (c).

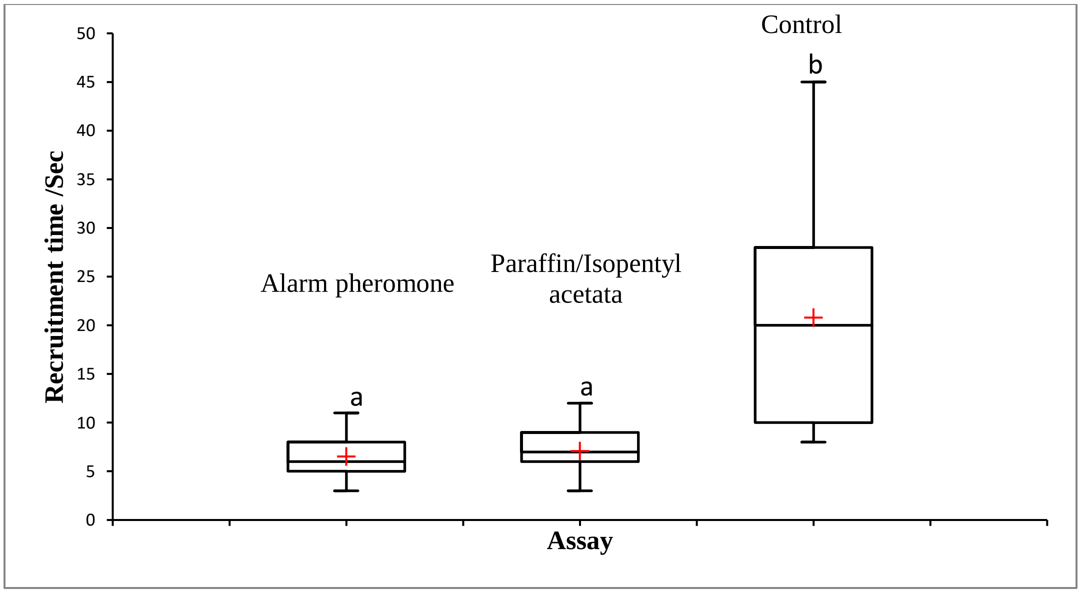

Figure S2: Box plot for the time of recruitment of honey bee per assay. Means with different small letter differ significantly among assays at $P < 0.05$, One-way ANOVA, Tukey (HSD) post-hoc test.

The time at which honey bees responded to the alarm pheromone assay was faster compared to paraffin mixed with isopentyl acetate while honey bees took more time to respond to the empty suedes.

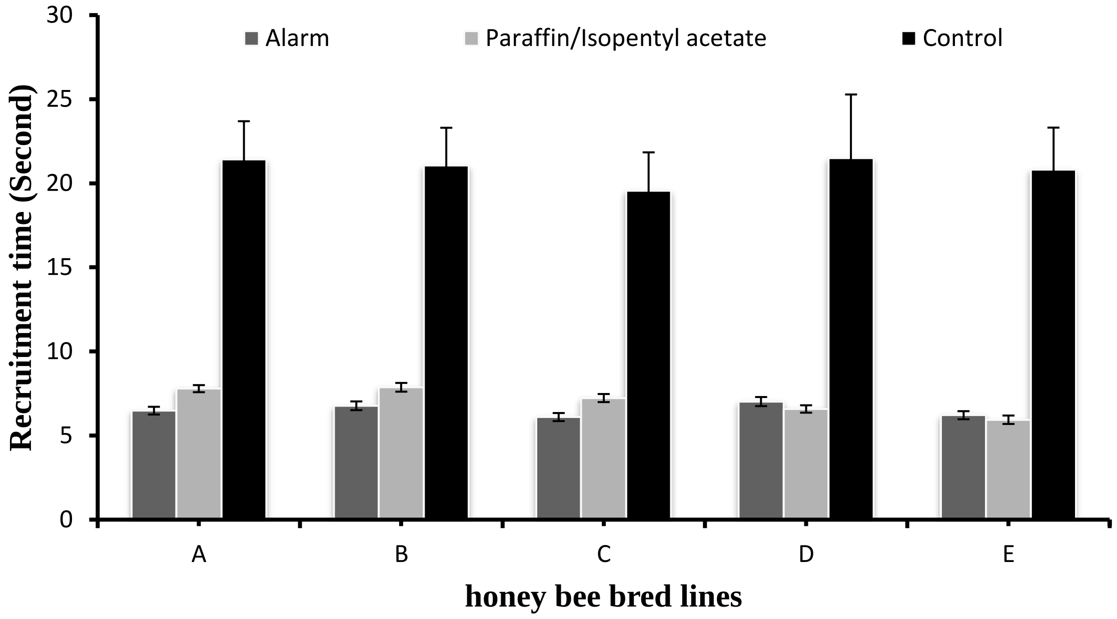

Figure S3: Variation in the recruitment time among bred lines of honey bee colonies.

Table S2: Variation in the mean number of stings among bred lines when colonies were struck with a standard marble stimulus and when colonies were not struck with a standard marble stimulus.

Bred lines	Marbled		U - value	P-value (Alarm vs Paraffin	Non-marbled		U - value	P-value (Alarm vs Paraffin
	Alarm pheromone (Mean ± SE)	Paraffin (Mean ± SE)			Alarm pheromone (Mean ± SE)	Paraffin (Mean ± SE)		
A	10.81 ± 1.69a	9.31 ± 1.76ab	1258	0.438	2.1 ± 0.44a	1.23 ± 0.36a	1387	0.063
B	10.9 ± 1.83a	8.37 ± 1.63a	1275	0.363	2.02 ± 0.44a	0.83 ± 0.21a	1456	0.016
C	27.06 ± 3.71b	15.5 ± 2.36ab	1467	0.021	4.42 ± 0.75ab	1.56 ± 0.41a	1611.5	0.0004
D	24.52 ± 3.26ab	19.6 ± 3.02b	1280	0.348	4.42 ± 0.95ab	1.56 ± 0.33a	1443.5	0.027
E	28.92 ± 3.71b	17 ± 2.4b	1432	0.04	6.48 ± 1.24b	4.23 ± 1.15a	1454	0.024

Means with different small letter in a column are significantly different among bred lines at P < 0.05, Kruskal-Wallis test using Dunn’s procedure.
